# Dissecting “Cotyledonoid” Leiomyoma Involved by Adenomyosis

**DOI:** 10.1177/10668969241308200

**Published:** 2025-01-08

**Authors:** Samuel Robichaud, Jahg Wong, Karim Ouallouche, Nathalie Bleau, Kurosh Rahimi

**Affiliations:** 1 Department of Pathology, 5622 Université de Montréal, Montreal, Quebec, Canada; 2 Departement of Pathology, 103380 Fleury Hospital, Montreal, Quebec, Canada; 3 Department of Obstetrics and Gynecology, 5622 University of Montreal, Montreal, Quebec, Canada

**Keywords:** dissecting leiomyoma, cotyledonoid leiomyoma, adenomyosis, adenosarcoma, Sternberg tumor

Uterine leiomyomas are routinely seen by surgical pathologists and generally pose little diagnostic issues despite their multiple morphologic subtypes. We however report a specimen of dissecting leiomyoma involved by adenomyosis, mimicking uterine adenosarcoma. This distinction is important to guide therapy as the latter is a malignant neoplasm.

We report a 29-year-old woman who presented with persisting menorrhagia and abdominal pain who underwent a pelvic ultrasound and MRI which showed a uterine mass with mixed solid and cystic components that was estimated to measure 11 by 7 cm. As a result, the patient underwent myomectomy.

The gross specimen consisted of a multinodular, tan-colored mass weighing 296 g, and measuring 13 by 7 by 8 cm. Overall the nodules had a smooth surface, though some of them protruded through the uterine serosa, imparting a grape-like or placental-like appearance. No areas of necrosis or hemorrhage were present. An intra-uterine device was also found.

**Figure 1.**
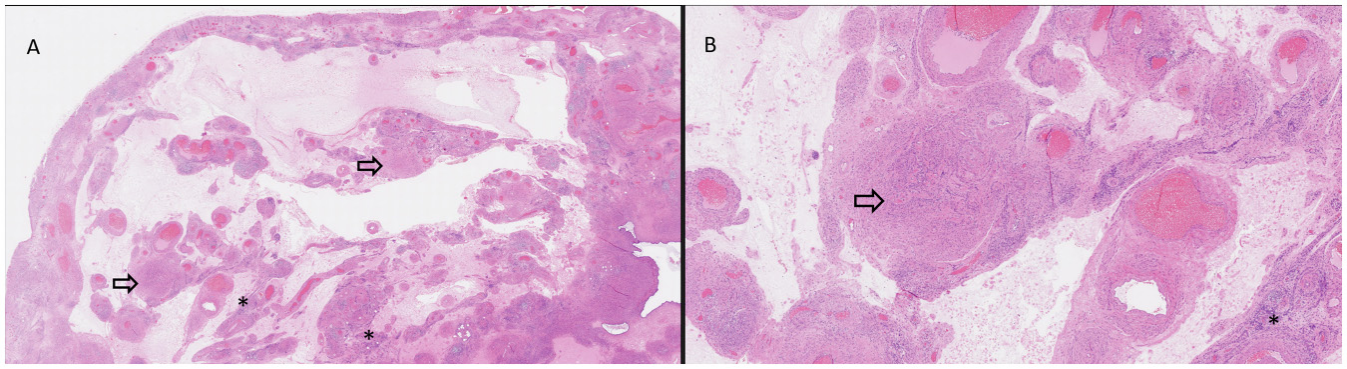
Histological examination of the uterine mass revealed lobular smooth muscle nodules (arrow) separated by zones of hydropic changes and thick-walled vessels (2A: hematoxylin and eosin [H&E], original magnification × 10; 2B: H&E, original magnification × 40) combined with foci of adenomyosis (asterisk).

Histopathologic analysis showed multiple eosinophilic normocellular nodules that irregularly dissected through surrounding myometrium that additionally showed marked hydropic changes. The nodules consisted of bland spindle cells with cigar-shaped nuclei with blunt ends and abundant eosinophilic cytoplasm, characteristic of smooth muscle cells. There was no prominent nuclear atypia or tumor necrosis. Mitotic figures were also absent to rare (under 2 per mm^2^). The spindle cell nodules were accompanied by thick-walled vessels. However, smooth muscle nodules were involved by irregular glandular structures lined by bland cuboidal epithelium without any convincing mucinous, ciliated, or morular differentiation. The glandular structures were confined to the smooth muscle nodules without invasion into the surrounding myometrial fibers. No vascular invasion was identified in either the glandular or the mesenchymal component. Of note, areas reminiscent of endometrial stroma were noted around the glandular structures (Figure 1).

**Figure 2.**
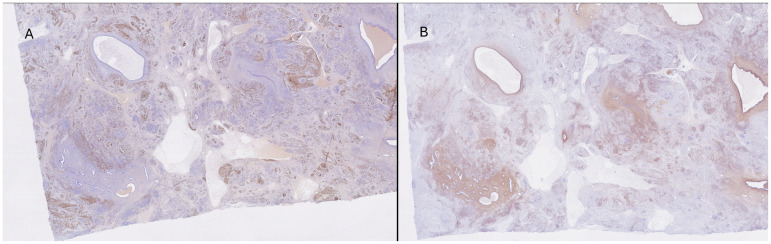
The smooth muscle nodules stained for caldesmon (3A: caldesmon, original magnification × 10; 3B: CD10, original magnification × 10) whereas the stroma of the adenomyosis component stained positive for CD10.

By immunohistochemical studies, the spindle cell component majoritarily forming the smooth muscle nodules was positive for caldesmon (CALD1), calponin (CNN) and desmin (DES). The epithelial component and the smooth muscle component showed a wild-type p53 pattern. Ki67 proliferation index was low in both components and was under 5%. CD10 (MME) was positive within the endometrial stromal cells surrounding glandular structures (Figure 2).

Our lesion showed a bland smooth muscle tumor with a dissecting cotyledoid pattern involved by glandular structures surrounded by endometrial stroma. Our main diagnostic consideration was a dissecting cotyledoid leiomyoma involved by adenomyosis, although the differential diagnosis included “biphasic” (epithelial and mesenchymal) tumors of the uterus such as adenosarcoma and uterine carcinosarcoma.

Elements in favor of a dissecting cotyledoid leiomyoma involved by adenomyosis include the confinement of the adenomyosis within the confines of the smooth muscle, without frank myometrial invasion or any other malignant features. Although areas of peri-glandular hypercellularity raised concern for the “cambium layer” characteristic of adenosarcoma, the lack of stromal atypia, leaf-like projections and “stromal overgrowth” argued against an adenosarcoma. Additionally, lack of high-grade atypia and mutated p53 (TP53) expression patterns argued against carcinosarcoma. Lack of squamous differentiation and glandular atypia akin to endometrial intraepithelial neoplasia argued against atypical polypoid adenomyomatous tumor.

Dissecting leiomyoma is a morphologic variant of uterine leiomyoma in which nodules of smooth muscle irregularly dissect the smooth muscles of the myometrium.^1^ When the smooth muscle nodules extend outside of the uterus they may impart a grape-like or a placental-like appearance of the serosal surface, often referred to as “cotylenodoid leiomyoma”.^2^ In our specimen, the irregular dissecting of the smooth muscle nodules gave an ostensively invasive pattern to the foci of adenomyoma. This co-occurrence has been described in previous reports, including a specimen with lung metastasis.^3,4^

Although leiomyomas and adenomyosis are routinely seen by surgical pathologists, our specimen illustrates that the irregular architecture of the dissecting cotyledoid leiomyoma, especially when involved by adenomyosis, may mimic other biphasic tumors of the uterus. This distinction has important management implications since leiomyomas and adenomyosis are benign entities, that only required medical or surgical intervention depending on symptoms.^5^ In contrast, adenosarcomas and carcinosarcomas are malignant neoplasms that require surgical resection and pathologic staging and may qualify for systemic therapy.^6^
